# Antimicrobial resistance among migrants in Europe: a systematic review and meta-analysis

**DOI:** 10.1016/S1473-3099(18)30219-6

**Published:** 2018-07

**Authors:** Laura B Nellums, Hayley Thompson, Alison Holmes, Enrique Castro-Sánchez, Jonathan A Otter, Marie Norredam, Jon S Friedland, Sally Hargreaves

**Affiliations:** a Department of Medicine, Imperial College London, London, UK; b Danish Research Centre for Migration Ethnicity and Health, University of Copenhagen, Copenhagen, Denmark; c Section of Immigrant Medicine, Department of Infectious Disease, Copenhagen University Hospital, Hvidovre, Denmark

## Abstract

**Background:**

Rates of antimicrobial resistance (AMR) are rising globally and there is concern that increased migration is contributing to the burden of antibiotic resistance in Europe. However, the effect of migration on the burden of AMR in Europe has not yet been comprehensively examined. Therefore, we did a systematic review and meta-analysis to identify and synthesise data for AMR carriage or infection in migrants to Europe to examine differences in patterns of AMR across migrant groups and in different settings.

**Methods:**

For this systematic review and meta-analysis, we searched MEDLINE, Embase, PubMed, and Scopus with no language restrictions from Jan 1, 2000, to Jan 18, 2017, for primary data from observational studies reporting antibacterial resistance in common bacterial pathogens among migrants to 21 European Union-15 and European Economic Area countries. To be eligible for inclusion, studies had to report data on carriage or infection with laboratory-confirmed antibiotic-resistant organisms in migrant populations. We extracted data from eligible studies and assessed quality using piloted, standardised forms. We did not examine drug resistance in tuberculosis and excluded articles solely reporting on this parameter. We also excluded articles in which migrant status was determined by ethnicity, country of birth of participants' parents, or was not defined, and articles in which data were not disaggregated by migrant status. Outcomes were carriage of or infection with antibiotic-resistant organisms. We used random-effects models to calculate the pooled prevalence of each outcome. The study protocol is registered with PROSPERO, number CRD42016043681.

**Findings:**

We identified 2274 articles, of which 23 observational studies reporting on antibiotic resistance in 2319 migrants were included. The pooled prevalence of any AMR carriage or AMR infection in migrants was 25·4% (95% CI 19·1–31·8; *I*^2^ =98%), including meticillin-resistant *Staphylococcus aureus* (7·8%, 4·8–10·7; *I*^2^ =92%) and antibiotic-resistant Gram-negative bacteria (27·2%, 17·6–36·8; *I*^2^ =94%). The pooled prevalence of any AMR carriage or infection was higher in refugees and asylum seekers (33·0%, 18·3–47·6; *I*^2^ =98%) than in other migrant groups (6·6%, 1·8–11·3; *I*^2^ =92%). The pooled prevalence of antibiotic-resistant organisms was slightly higher in high-migrant community settings (33·1%, 11·1–55·1; *I*^2^ =96%) than in migrants in hospitals (24·3%, 16·1–32·6; *I*^2^ =98%). We did not find evidence of high rates of transmission of AMR from migrant to host populations.

**Interpretation:**

Migrants are exposed to conditions favouring the emergence of drug resistance during transit and in host countries in Europe. Increased antibiotic resistance among refugees and asylum seekers and in high-migrant community settings (such as refugee camps and detention facilities) highlights the need for improved living conditions, access to health care, and initiatives to facilitate detection of and appropriate high-quality treatment for antibiotic-resistant infections during transit and in host countries. Protocols for the prevention and control of infection and for antibiotic surveillance need to be integrated in all aspects of health care, which should be accessible for all migrant groups, and should target determinants of AMR before, during, and after migration.

**Funding:**

UK National Institute for Health Research Imperial Biomedical Research Centre, Imperial College Healthcare Charity, the Wellcome Trust, and UK National Institute for Health Research Health Protection Research Unit in Healthcare-associated Infections and Antimictobial Resistance at Imperial College London.

## Introduction

Antimicrobial resistance (AMR) is increasing worldwide, presenting substantial challenges to the prevention and treatment of common bacterial infections.^1^, ^2^ This resistance results in worse and more costly health outcomes^3^, ^4^ and an increased risk of morbidity and mortality.^5^, ^6^, ^7^ In Europe, resistance has been reported for every major class of antibiotic in both community and health-care settings.^8^ Various factors might be contributing toward onward transmission of resistance, including travel, migration, and socioeconomic factors.^9^, ^10^, ^11^, ^12^, ^13^

In Europe, combined resistance to fluoroquinolones, third-generation cephalosporins, and aminoglycosides increased between 2011 and 2014 in isolates for Gram-negative bacteria, including *Klebsiella pneumoniae* (from 16·7% to 19·6%) and *Escherichia coli* (from 3·8% to 4·8%).^6^ In England, bloodstream infections caused by *E coli* resistant to the most frequently used antibiotics for sepsis (eg, piperacillin–tazobactam) increased from 8·5% to 11·7% between 2011 and 2015; resistant *K pneumoniae* increased from 12·6% to 18·5% in the same period.^14^ These changes will result in an increased reliance on carbapenems, which are considered as antibiotics of last resort, and there are concerns about rising rates of carbapenem-resistant bacteria worldwide. Although the prevalence of meticillin-resistant *Staphylococcus aureus* (MRSA) in tested isolates decreased slightly in Europe during this period (from 18·6% to 17·4%), it remains high in some parts of Europe and worldwide. For example, the prevalence of MRSA is 0·3–60% in Europe, 12–89% in Africa, and 10–53% in eastern Mediterranean countries, from which many migrants to Europe originate.^6^

**Research in context**

**Evidence before this study**

Rates of antimicrobial resistance (AMR) are rising in Europe, and there is concern that increased migration, especially among refugees and asylum seekers, might be contributing to the burden of antibiotic resistance. Following PRISMA guidelines, we searched MEDLINE, Embase, PubMed, and Scopus, using terms pertaining to migration, antibiotic resistance, common bacterial infections, and European Union (EU)-15 and European Economic Area (EEA) countries, for articles published between Jan 1, 2000, and Jan 18, 2017. Search terms are given in the appendix. Articles reporting primary research on antibiotic resistance in key common bacteria in migrants were included with no language restrictions. There is insufficient evidence regarding patterns of AMR in migrants to Europe. As a result, the size of the burden of AMR is in these populations, or where drug-resistant organisms are acquired, remain unknown. Infection prevention and control responses require a robust and comprehensive understanding of the scientific literature, and therefore systematic reviews and meta-analyses are needed in this area to develop robust evidence-based strategies to counter AMR. We did this systematic review and meta-analysis to identify and synthesise data on antibiotic resistance carriage or infection in migrants to countries in the EU, EEA, and Switzerland to examine differences in patterns of AMR across migrant groups and in high-migrant community settings.

**Added value of this study**

The relationship between AMR and migration to Europe has not previously been systematically examined. Given that migration and antibiotic resistance are increasing in Europe, robust evidence is needed to inform policy and practice to improve the prevention, detection, and treatment of AMR. This systematic review and meta-analysis examines data on antibiotic resistance among 2319 migrants to EU-15 and EEA countries reported in 23 articles. Our findings show that the pooled prevalence of any antibiotic resistance carriage or infection was increased in refugees and asylum seekers compared with other migrants, and in high-migrant community settings rather than in hospitals. There was also evidence that antibiotic-resistant organisms are being acquired during and following migration, with little evidence of onward transmission to host populations.

**Implications of all the available evidence**

Migrants—refugees and asylum seekers in particular—are exposed to conditions (eg, overcrowding, poor sanitation, and poor access to health services) in transit and host countries in Europe that favour the emergence of AMR. The evidence suggests that migrants acquire drug-resistant bacteria in high-migrant community settings, such as refugee camps, transit centres, or detention facilities in host countries, with little evidence that there is substantial onward transmission to local European host populations. These findings highlight that migrant communities are vulnerable to exposure to AMR in Europe and that initiatives aiming for improved prevention, detection, and treatment of antibiotic-resistant infections in high-migrant community settings, supported by better social conditions and access to health services, are urgently needed for these migrant communities.

More than 30 million European Union (EU) residents are born outside the EU,^15^ and more than 2 million migrants have entered Europe since 2015 during the recent refugee crisis.^16^ Whether migrants—and refugees and asylum seekers in particular—have high rates of AMR carriage or infection, or where bacterial resistance could be acquired, remains unknown. Some migrant groups might have an increased risk of antibiotic resistance because of poor sanitation or overcrowded living conditions, barriers to accessing health services, or disruptions in treatment during migration and on arrival in host countries.^17^ Additionally, high prevalence rates of AMR in countries of origin or transit associated with insufficient health infrastructure, antibiotic stewardship, infection prevention and control, vaccine coverage, or surveillance—particularly in the context of conflict—could increase the risk of bacterial resistance, as could return travel to their countries of origin and contact with health services in these settings.^10^, ^18^, ^19^ Alternatively, the risk of antibiotic resistance in migrant communities might be lower than it is in locally born populations in Europe because of reduced access and exposure to antibiotics or health-care facilities. Thus, rather than importing antibiotic resistance, migrants could instead be vulnerable to exposure to AMR pathogens in host countries.

The absence of data on AMR carriage or infection in migrants to Europe might be partly attributable to insufficient surveillance in migrants' countries of origin and within migrant populations in host countries.^6^ For example, 70% of migrants arriving in Europe during the recent refugee crisis originated from Afghanistan, Eritrea, Iraq, Nigeria, and Syria.^20^ Surveillance data for priority antibiotic-resistant organisms in these countries are unavailable, athough incidence is high in neighbouring countries for which data are available.^21^ For example, for *E coli*, reported resistance to third-generation cephalosporins is 19–33% in Lebanon, 23–31% in Jordan, and 10–94% in Pakistan.^6^ Data on AMR carriage or infection are also insufficient because of variations in approaches to surveillance and screening across EU countries, which are mostly done in secondary and tertiary health-care settings (eg, hospitals) rather than in community settings, such as primary care. This approach further restricts the availability of data about AMR in migrant populations in view of the barriers they experience in accessing health services^22^ and fails to capture risk factors favouring the emergence of drug resistance in settings like refugee camps, transit centres, or detention facilities.^23^, ^24^ Consequently, the epidemiology of AMR in migants, and extent to which high rates of migration might contribute to the burden of antibiotic resistance in Europe, remain unclear.^11^, ^25^, ^26^ Although infection prevention and control strategies rely on published scientific literature, to our knowledge, peer-reviewed primary research reporting AMR in migrant populations has not been comprehensively examined. Systematic reviews and meta-analyses are essential for transparently and systematically summarising available evidence,^27^, ^28^ and are needed in this field to develop robust and evidence-based responses in policy and practice.

The aim of this systematic review and meta-analysis was to identify and synthesise data on AMR, including carriage of or infection with antibiotic-resistant organisms, in migrants to countries in the EU, European Economic Area (EEA), and Switzerland to examine differences in patterns of antibiotic resistance across migrant groups, such as refugees and asylum seekers, and in high-migrant community settings, such as refugee camps and hospitals.

## Methods

### Search strategy and selection criteria

For this systematic review and meta-analysis, we did a literature search to identify peer-reviewed articles reporting primary research from observational studies in EU-15 and EEA countries and Switzerland to capture 21st Century patterns of migration and antibiotic-resistant organisms. The study conforms to the Preferred Reporting Items for Systematic reviews and Meta-Analysis (PRISMA) guidelines.^29^

Two reviewers (LBN and HT) searched MEDLINE, Embase, PubMed, and Scopus for peer-reviewed articles reporting primary research from observational studies done between Jan 1, 2000, and Jan 18, 2017. We used a Boolean search strategy with search terms pertaining to migration, antibiotic resistance, the common bacterial infections of interest, EU-15 and EEA countries, and the appropriate MeSH headings for each database. Search terms were identified from relevant research, systematic reviews, reports, experts in migrant health and AMR, and the 2014 WHO report on the surveillance of antimicrobial resistance.^6^, ^30^, ^31^, ^32^, ^33^ A detailed summary of all search terms is provided in the appendix.

To be eligible for inclusion, articles had to report carriage or infection with laboratory-confirmed antibiotic-resistant organisms in migrants. In our analysis, AMR carriage or infection includes MRSA and Gram-negative bacteria, including extended-spectrum β-lactamase (ESBL)-producing bacteria or multidrug-resistant bacteria, with combined resistance defined as resistance to three or more antimicrobial groups. Migrants were defined as individuals born outside the country in which the study was conducted, including refugees, asylum seekers, and other migrant groups. An individual was categorised as a refugee or asylum seeker where these terms were used to classify them in the relevant article. Refugees and asylum seekers were defined as individuals who have been granted asylum or are seeking or have previously sought asylum under the 1951 UN Convention on the Status of Refugees. Other migrants were all other foreign-born individuals who were not categorised as refugees or asylum seekers. Other reasons for migration included family, work, or education.

This systematic review and meta-analysis focuses on antibiotic resistance in common bacterial pathogens^6^ and the evidence gap around its magnitude in migrant populations. We did not examine drug resistance in tuberculosis and excluded articles solely reporting on this factor. We also excluded articles in which migrant status was determined by ethnicity, country of birth of participants' parents, or was not defined, and articles in which data were not disaggregated or reported by migrant status. Comments, editorials, reviews, letters, case reports, and duplicate studies were also excluded. No language restrictions were placed on the searches or search results. Non-English articles were included and translated before full-text screening.

Two reviewers (LBN and HT) manually screened the bibliographies of included articles to identify additional eligible studies. In cases where articles could not be accessed, authors were contacted to retrieve the full texts. Title and abstract screening, full text screening, data extraction, and quality assessment were also done independently by LBN and HT. Any discrepancies were discussed until a consensus was reached. EndNote X7 and Rayyan programmes were used for screening. Piloted forms were used for data extraction and quality assessment. Summary data were extracted on study design, country, screening setting, recruitment, population, sample size, sample demographics, period of study, and reported AMR carriage or infection. The quality assessment form comprised relevant components of validated quality assessment and risk of bias frameworks including the Critical Appraisal Skills Programme tools, GRADE,^34^ the Newcastle-Ottawa Scale,^35^ and the Cochrane Effective Practice and Organisation of Care Group.^36^ Articles were given a quality score percentage to reflect methodological rigour (for relevant study design) and clarity and transparency in reporting. We did not exclude articles based on quality scoring; however, sensitivity analyses were conducted. The quality assessment is available on request.

### Data analysis

Articles that met the inclusion criteria and reported prevalence data for AMR in migrant (foreign-born) populations were included in the meta-analysis. Articles identified through the literature search that reported on AMR in migrants but that did not report prevalence data were not included in the meta-analysis. To adjust for heterogeneity across the studies (eg, across settings or migrant populations), we used random-effects models for the analyses. Pooled estimates of the prevalence of any detected AMR carriage or infection were calculated for all migrants across the included articles; by migrant status for refugees and asylum seekers and other migrant groups; and by setting for high-migrant community settings (eg, camps, transit centres, detention centres, or reception centres) and hospital settings. We did subanalyses for MRSA and drug-resistant Gram-negative bacteria. We also calculated pooled estimates of the prevalence of AMR carriage and AMR infection in migrants across the included articles. We used sensitivity analyses to examine the effect of quality across the articles. Heterogeneity was explored graphically in forest plots to determine whether characteristics such as sex, study country, date of data collection, country of origin, or screening approach across the studies might explain sources of heterogeneity, and in stratified analyses in relation to migrant status, setting, and infection versus carriage.

Data were analysed using Stata 14. Metaprop, a Stata command, was used to calculate pooled estimates for prevalence and 95% CIs.^37^ In the meta-analyses, a continuity correction of 0·5 was added to zero cells. Heterogeneity was assessed through the use of the *I*^2^ statistic.^38^ This systematic review and meta-analysis is registered with PROSPERO, number CRD42016043681.

### Role of the funding source

The funders had no role in study design, data collection, data analysis, data interpretation, or writing of the report. The corresponding author had full access to all the data in the study and had final responsibility for the decision to submit for publication.

## Results

**Figure 1:**
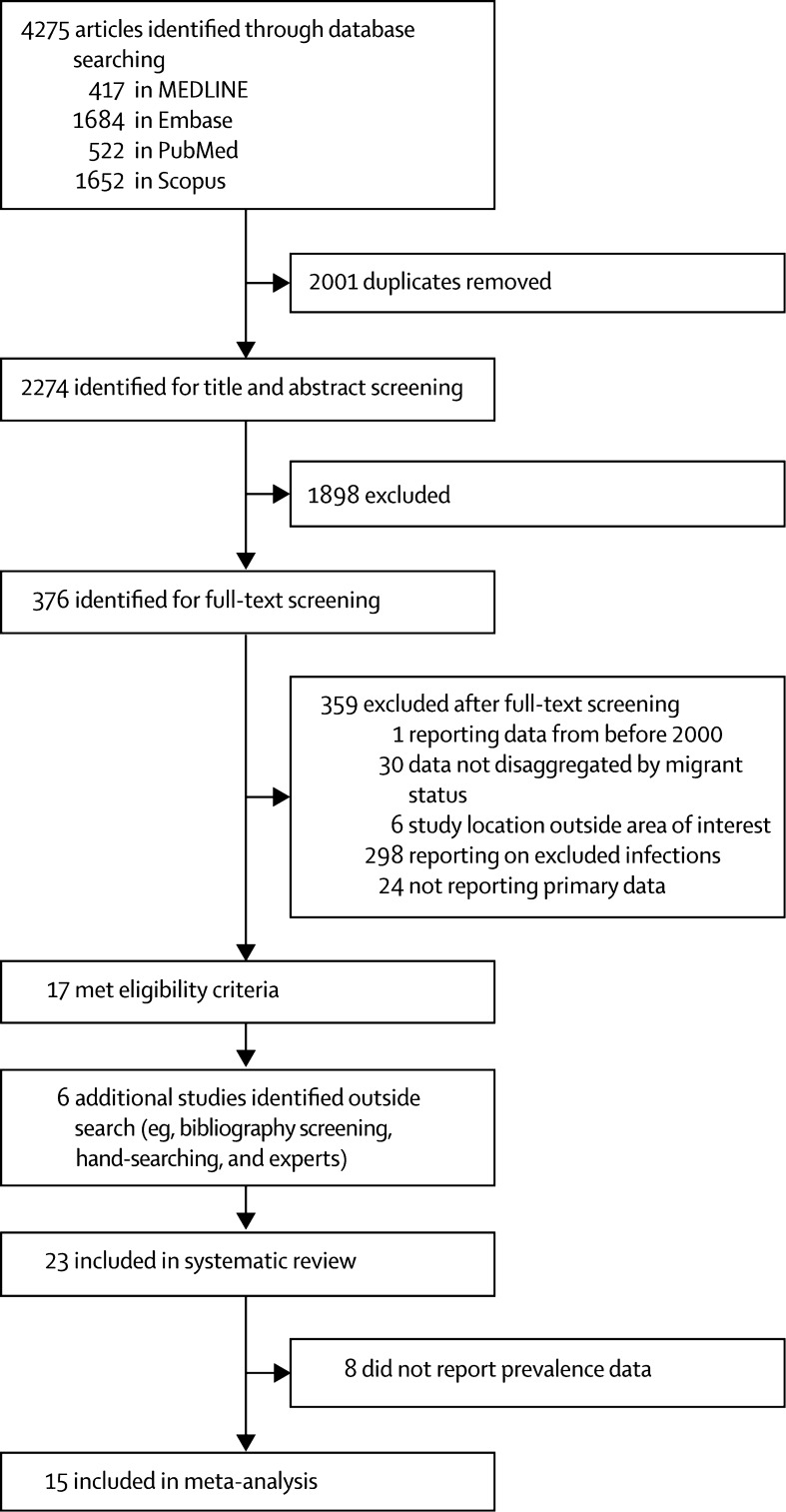
Study selection

4275 reports were identified in the database search. After removal of duplicates, 2274 articles were screened for eligibility, of which 376 were included in the full-text screening (figure 1). 23 articles^39^, ^40^, ^41^, ^42^, ^43^, ^44^, ^45^, ^46^, ^47^, ^48^, ^49^, ^50^, ^51^, ^52^, ^53^, ^54^, ^55^, ^56^, ^57^, ^58^, ^59^, ^60^, ^61^ reporting on antibiotic resistance in 2319 migrants met the inclusion criteria and were included in the systematic review; 15 articles reporting prevalence data on AMR in migrants were included in the main meta-analysis (table 1).

**Table 1:** Characteristics of included studies

	**Country**	**Study details**	**Number of migrants**	**Prevalence of MDRO**	**MRSA**		**Drug-resistant Gram-negative bacteria**					**Number of people infected**	**Number of carriers**	**Quality assessment (%)**†
					Prevalence	Migrants as proportion of PVL-positive MRSA	ESBL or combined resistance*	Combined resistance*	ESBL *Escherichia coli*	ESBL *Klebsiella pneumoniae*	ESBL shigella			
Angeletti et al (2016)^39^	Italy	Syrian asylum seekers (median age 20 years) screened at an asylum seeker centre on arrival; rectal, pharyngeal, and nasal swabs were collected	48	12 (25·0%, 12·8–37·3)	6 (12·5%, 3·1–21·9)	..	6 (12·5%, 3·1–21·9)	..	4 (8·3%, 0·5–16·1)	2 (4·2%, 0·1–9·9)	..	..	12	60·7
Broseta et al (2006)^40^	Spain	Migrant patients (aged <16 years) admitted to one hospital who tested positive for MRSA; PVL-positive MRSA included skin or soft tissue infections, otitis, and bacteraemic pyomiositis	4	4 (100%)	4 (100%)	57·1%	..	..	..	..	..	..	..	88·5
Casado-Verrier et al (2012)^41^	Spain	Migrant patients (mean age 40 years [SD 16·8]) with a community-acquired skin or soft tissue infection who attended a hospital emergency department and were screened for MRSA; clinical, microbiological, and epidemiological data were collected	8	8 (100%)	8 (100%)	54·5%	..	..	..	..	..	..	..	96·9
Cercenado et al (2008)^42^	Spain	Migrant patients (aged 2 days to 34 years) who attended an emergency department and were screened for MRSA; MRSA strains resistant to three or more antimicrobial groups of drugs were screened for PVL; epidemiological features of PVL-positive cases: skin and soft tissue infection, acute otitis media, bacteraemia, and pharyngeal and nasal colonisation	7	7 (100%)	7 (100%)	53·8%	..	..	..	..	..	..	..	88·5
Dudareva et al (2011)^43^	Germany	Residents (mean age 22·7 years [SD 14·1]) of an asylum seeker centre screened using convenience sampling; nasal, throat, axilla, and groin swabs collected; data collected about community-acquired skin and soft tissue infections	232	5 (2·2%, 0·3–4·1)	5 (2·2%, 0·3–4·1)	..	..	..	..	..	..	..	5	78·6
Frick et al (2010)^44^	Spain	Child migrants (aged <16 years) positive for community-associated MRSA after testing in a hospital's paediatrics department; data collected for community-acquired skin and soft tissue infections, humeral osteomyelitis associated with pneumonia, and bacteraemia	6	6 (100%)	6 (100%)	50·0%	..	..	..	..	..	..	..	96·2
Georgakopoulou et al (2016)^45^	Greece	Detection of shigella infection in refugee children (mean age 4·7 years [SD 3·5]) after implementation of a syndromic notification system in one transit centre in Athens; isolates tested for resistance	16; 13 isolates tested	7 (53·8%, 26·7–80·9)	..	..	7 (53·8%, 26·7–80·9)	..	..	..	7 (53·8%, 26·7–80·9)	7	..	73·1
Gustafsson et al (2006)^46^	Sweden	Adopted migrant children (aged 3–16 years) with a history of hospital admission or contact in home country screened for MRSA on arrival to University Hospital Lund along with children who arrive from institutions where a high prevalence of MRSA is suspected; swabs of anterior nares, throat, perineum, and skin lesions collected; clinical MRSA infection found in four children with infected skin lesions	23	13 (56·5%, 36·2–76·8)	13 (56·5%, 36·2–76·8)	..	..	..	..	..	..	4	13	78·6
Hagleitner et al (2012)^47^	Netherlands	All adopted migrant children (aged 0·5–7·5 years) undergoing health examination and MRSA screening on arrival; swabs of anterior nares, throat, perineum, and wounds collected—no clinical MRSA infections were identified	131	17 (13·0%, 7·2–18·8)	17 (13·0%, 7·2–18·8)	..	..	..	..	..	..	0	17	84·2
Heudorf et al (2016)^48^	Germany	Unaccompanied refugee minors (aged <18 years) upon arrival at an asylum seekers' centre were screened for multidrug-resistant Gram-negative bacteria carriage; stool samples were collected	119	42 (35·3%, 26·7–43·9)	..	..	42 (35·3%, 26·7–43·9)	9 (7·6%, 2·8–12·4)	37 (19·3%, 14·5–24·9)	5 (4·2%, 0·6–7·8)	..	0	42	83·3
Heudorf et al (2016)^49^	Germany	Refugee (adults and children) hospital admissions reported by hospitals in the Frankfurt Rhine-Main region of Germany to the public health department	MRSA screening: 325; MRGN screening: 290	..	32 (9·8%, 6·6–13	..	67 (23·1%, 18·3–28·0)	24 (8·3%, 5·1–11·5); ESBL-positive: 37 (12·8%, 9·0–16·7)	..	..	..	..	..	71·4
Krüger et al (2016)^50^	Germany	Refugee children (aged 0–17 years) and pregnant women residing in camps admitted to one hospital; nasopharyngeal and rectal swabs were collected; urinary tract infections found; no other clinical manifestations	62 children; 11 pregnant women	15 children, 3 women: 18 (24·7%, 14·8–34·6)	10 children, 1 woman: 11 (15·1%, 6·9–23·3)	..	8 (11%, 3·8–18·2)	6 children, 2 women: 8 (11%, 3·8–18·2)	..	..	..	1	18	94·1
Lederer et al (2015)^51^	Austria	Shigella screening in refugees (aged 1–65 years) implemented during patient consultations at medical care facilities in transit centres (passive surveillance) and during entry health examination at asylum seeker reception centres (active compulsory screening for asylum seekers)	15	9 (60·0%, 35·2–84·8)	..	..	9 (60·0%, 35·2–84·8)	..	..	..	8 (53·3%, 28·1–78·6)	9	..	61·5
Manzur et al (2007)^52^	Spain	Positive MRSA isolates from screening or clinical samples from migrants (aged 10–69 years) in a university hospital setting tested for PVL; patient data were acquired retrospectively from clinical files; specific focus on community-acquired MRSA	15	15 (100%)	15 (100%)	78·9%	..	..	..	..	..	..	..	76·7
Marschall et al (2006)^53^	Switzerland	Screening of pregnant migrant women (aged 18–40 years) from former Yugoslavia in obstetric outpatient clinic in a university hospital; nasal and vaginal swabs collected	152	0	0	..	..	..	..	..	..	..	0	91·7
Oliva et al (2013)^54^	Italy	Retrospective study of HIV-positive patients and seronegative migrants (median age 45 years [IQR 39–49]) attending the outpatients clinic at the Department of Infectious and Tropical Diseases at the University of Rome screened for MRSA carriage; nasal swabs collected	96	0	0	..	..	..	..	..	..	..	0	73·5
Piso et al (2017)^55^	Switzerland	Cross-sectional rates of colonisation in four cantonal refugee centres in northwestern Switzerland in refugees (aged ≥15 years); throat, nasal, groin, and rectal swabs collected; skin and soft tissue infections were a risk factor for colonisation with MRSA	261 (MRSA); 241 (Gram-negative bacteria)	..	41 (15·7%, 11·8–20·6)	..	57 (23·6%, 18·7–29·4)	..	57 (23·6%, 18·7–29·4)	..	..	..	..	94·1
Ravensbergen et al (2015)^56^	Netherlands	Asylum seekers (median age 24 years [IQR 15–33]) admitted to hospital, the emergency department, or tuberculosis wards identified through unique insurance numbers and screened for drug-resistant Gram-negative bacteria and MRSA; nose, throat, rectum, and perineum swabs collected	130	40 (30·8%, 22·9–38·7)	10 (7·7%, 3·1–12·3)	..	30 (23·1%, 15·9–30·6)	..	20 (15·4%, 9·2–21·6)	4 (3·1%, 0·1–6·1)	..	..	40	94·1
Reinheimer et al (2016)^57^	Germany	Refugee patients (aged 1–65 years) admitted to the University Hospital Frankfurt am Main from refugee accommodation were screened on arrival for drug resistant Gram-negative bacteria and MRSA; rectal and nasal swabs collected	143	92 (64·3%, 56·5–72·2)	8 (5·6%, 2·5–10·7)	..	84 (58·7%, 50·6–66·8)	..	72 (50·3%, 42·1–58·5)	12 (8·4%, 3·9–13·0)	..	..	92	91·7
Steger et al (2016)^58^	Germany	Asylum seekers (mean age 25 years for men, 32 years for women) treated at hospital; nasal and rectal swabs collected; skin and soft tissue infections found	108; 105 tested: 99 tested for MRGN and ESBL; 96 tested for MRSA	12 (11·4%, 5·3–17·5)	4 (4·2%, 0·2–8·2)	..	8 (8·1%, 2·7–13·5)	6 (6·1%, 1·4–10·8)	8 (8·1%, 2·7–13·5)	..	..	2	12	91·7
Stenhem et al (2010)^59^	Sweden	National surveillance data of confirmed MRSA infection in migrants (adults and children) supplemented with patient information	36	36 (100%)	36 (100%)	..	..	..	..	..	..	..	..	94·4
Tenenbaum et al (2016)^60^	Germany	Paediatric refugees (aged 1–202 months) admitted to a university hospital; rectal and nasopharyngeal swabs collected for MRGN and MRSA in all patients; skin and soft tissue infections and pyelonephritis found	325	110 of 325 (33·8%, 28·9–39·2)	22 of 325 (6·8%, 4·5–10·0)	..	..	26 of 136 (19·1%, 13·4–26·5)	68 of 325 (20·9%, 16·9–25·7)	18 of 325 (5·5%, 3·5–8·6)	..	4	110	92·3
Valverde et al (2015)^61^	Spain	Travellers and migrants (median age 33·5 years [range 29–40]) who attended the Tropical Medicine Unit at Ramón y Cajal University Hospital; stool samples collected	11	3 (27·3%, 1·0–53·6)	..	..	3 (27·3%, 1·0–53·6)	..	2 (18·2%, −6·3–10·3)	1 (9·1%, −7·9–26·1)	..	..	3	71·4

Data are n (%, 95% CI) unless otherwise stated. MDRO=multidrug-resistant organisms. MRSA=meticillin-resistant *Staphylococcus aureus*. PVL=Panton-Valentine leucocidin. ESBL=extended-spectrum β-lactamase. MRGN=multi-resistant Gram-negative bacteria.

* Combined resistance is resistance to three or more antimicrobial groups (eg, fluoroquinolones, third-generation cephalosporins, and aminoglycosides).

† Calculated by use of the piloted quality assessment form comprised of relevant validated tools for each study type.

**Figure 2:**
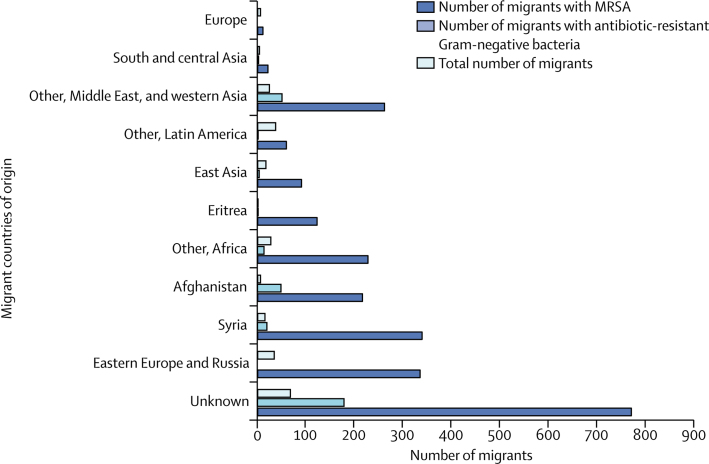
Distribution of antibiotic-resistant organisms among migrants by country of origin MRSA=meticillin-resistant *Staphylococcus aureus*.

Seven studies^43^, ^48^, ^49^, ^50^, ^57^, ^58^, ^60^ were done in Germany, six in Spain,^40^, ^41^, ^42^, ^44^, ^52^, ^61^ one in Greece,^45^ two in the Netherlands,^47^, ^56^ one in Austria,^51^ two in Sweden,^46^, ^59^ two in Switzerland,^53^, ^55^ and two in Italy.^39^, ^54^ Four articles were published in Spanish and translated before full-text screening.^40^, ^41^, ^42^, ^44^ The migrants' countries of origin across the studies are shown in figure 2. Of the 2319 migrants included across the studies, 1795 (77%) were asylum seekers or refugees (defined as those who have applied for or been granted asylum).^48^, ^49^, ^50^, ^51^, ^55^, ^56^, ^57^, ^58^, ^60^ Other migrants across the studies were non-refugee or asylum-seeking foreign-born individuals (eg, those migrating for economic or family reasons),^40^, ^41^, ^42^, ^52^, ^54^, ^59^, ^61^ including child migrants or adopted children.^44^, ^46^, ^47^ However, reason for migration was often not reported for individuals classified as other migrants, for whom only data on foreign-born status were available.

17 studies^40^, ^41^, ^42^, ^44^, ^46^, ^47^, ^49^, ^50^, ^52^, ^53^, ^54^, ^56^, ^57^, ^58^, ^59^, ^60^ were done in hospitals, six studies^39^, ^43^, ^45^, ^48^, ^51^, ^55^ were done in high-migrant community settings (eg, refugee camps or transit centres), and all but four^47^, ^49^, ^55^, ^59^ of the included studies were completed at a single site (table 1). The articles identified in the systematic review included studies reporting cohort data on AMR carriage or infection in migrants (not included in the meta-analysis),^40^, ^41^, ^42^, ^44^, ^52^, ^59^ or prevalence data on AMR carriage or infection in migrants identified through screenings targeted by specific population (eg, refugee or asylum seeker) or setting (eg, arrival at refugee centre or admission to hospital).

Antibiotic susceptibility testing methods for clinical and screening specimens across the studies and guidelines used to interpret antimicrobial sensitivity and minimum inhibitory concentrations are shown in the panel. Antibiotic susceptibility tests were performed for fluoroquinolones, aminoglycosides, carbapenems, and cephalosporins. 19 studies reported on MRSA,^39^, ^40^, ^41^, ^42^, ^43^, ^44^, ^46^, ^47^, ^49^, ^50^, ^52^, ^53^, ^54^, ^55^, ^56^, ^57^, ^58^, ^59^, ^60^ seven of which reported on community-associated MRSA or Panton-Valentine leucocidin expression, which is used as a surrogate for community-associated MRSA in the scientific literature.^40^, ^41^, ^42^, ^43^, ^44^, ^52^, ^55^ 12 studies reported on antibiotic resistance in Gram-negative bacteria.^39^, ^45^, ^48^, ^49^, ^50^, ^51^, ^55^, ^56^, ^57^, ^58^, ^60^, ^61^ Clinical manifestations, when reported, were predominantly skin and soft tissue infections or diarrhoea (table 1). Reporting was good for all key quality indicators and overall study quality was high (table 1).

Panel **Antibiotic susceptibility testing methods and guidelines**

**Antibiotic susceptibility testing methods**

• Disk diffusion^39^, ^40^, ^41^, ^42^, ^44^, ^45^, ^50^, ^52^, ^55^, ^61^
• Vitek 2^39^, ^48^, ^49^, ^50^, ^54^, ^55^, ^56^, ^57^, ^58^, ^60^
• E-test^42^, ^50^, ^51^, ^56^, ^57^
• Other methods^43^, ^46^, ^47^, ^48^, ^50^, ^53^, ^55^, ^58^, ^59^, ^61^

**Guidelines**

• Clinical & Laboratory Standards Institute Guidelines^39^, ^40^, ^41^, ^42^, ^49^, ^52^, ^57^, ^60^
• Guidelines from the European Committee on Antimicrobial Susceptibility Testing Steering Committee^39^, ^42^, ^45^, ^55^, ^56^, ^61^
• National guidelines^39^, ^46^, ^47^, ^51^, ^57^, ^59^

**Figure 3:**
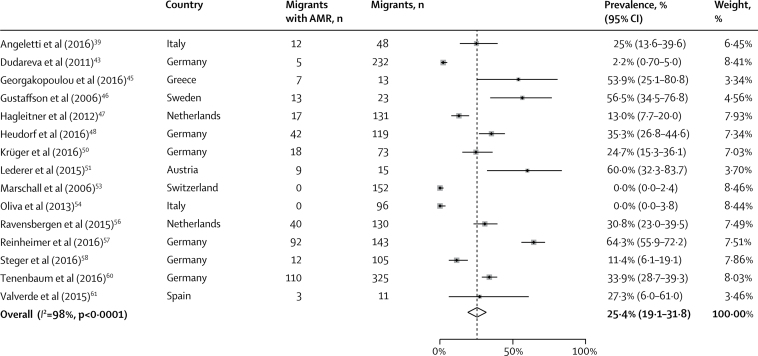
Pooled prevalence of antibiotic resistance among migrants to Europe AMR=antimicrobial resistance.

Overall, the pooled prevalence of any detected AMR carriage or infection across all migrants was 25·4% (95% CI 19·1–31·8; *I*^2^ =98%; figure 3), including MRSA (7·8%, 4·8–10·7; *I*^2^ =93%) and drug-resistant Gram-negative bacteria (27·2%, 17·6–36·8; *I*^2^ =94%; table 2), with variation in reported rates of AMR carriage or infection by country of origin (figure 2). The pooled prevalence of AMR infection across migrants in the included studies was 3·0% (0·0–5·9; *I*^2^ =87%), and the pooled prevalence of AMR carriage was 23·0% (16·5–29·4; *I*^2^ =98%; appendix). Reporting of AMR infection versus carriage across the articles was a source of heterogeneity, for which the *I*^2^ value reduced to 87% (appendix). Graphical explorations of heterogeneity did not suggest that other characteristics were significant sources of heterogeneity (data not shown).

**Table 2:** Antibiotic resistance across migrant groups and settings

	**All migrants**	**Refugees and asylum seekers**	**Other migrants**	**High-migrant community setting**	**Hospital**
Any detected antimicrobial resistance carriage or infection	25·4% (19·1–31·8)	33·0% (18·3–47·6)	6·6% (1·8–11·3)	33·1% (11·1–55·1)	24·3% (16·1–32·6)
Meticillin-resistant *Staphylococcus aureus*	7·8% (4·8–10·7)	8·2% (5·0–11·3)	6·0% (1·3–10·7)	9·8% (0·0–20·3)	7·4% (4·2–10·6)
Drug-resistant Gram-negative bacteria	27·2% (17·6–36·8)	27·2% (17·2–37·1)	27·3% (6·0–6·1)	32·1% (19·9–44·4)	24·9% (10·9–39·0)

Data are pooled prevalence (95% CI).

We did a sensitivity analysis to examine the effect of article quality on estimated prevalence of AMR carriage or infection across the included articles. When we excluded articles with a quality score of 75% or lower (25·9%, 95% CI 16·6–35·2), the pooled prevalence of AMR did not differ substantially from when we included all studies (25·4%, 19·1–31·8).

**Figure 4:**
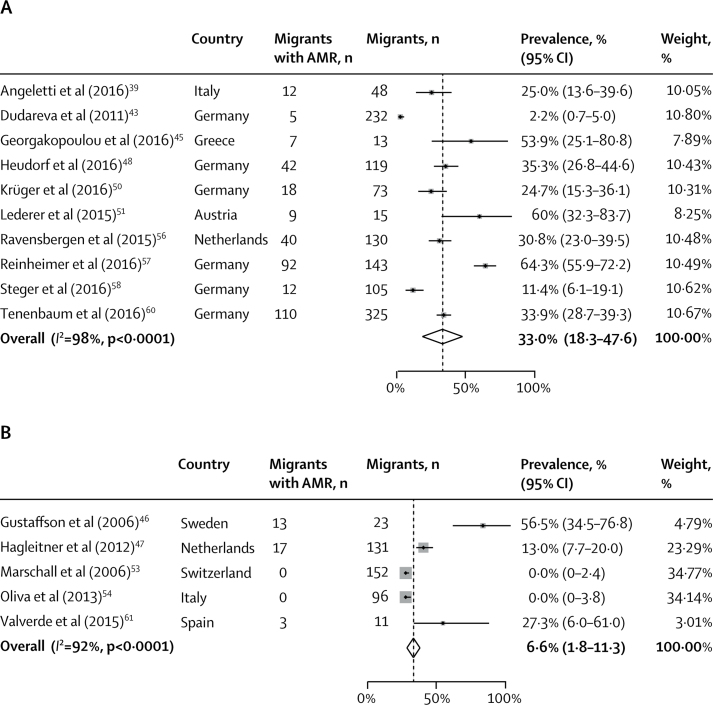
Pooled prevalence of antibiotic resistance by migrant type Prevalence among refugees and asylum seekers (A) and among other migrants (B). AMR=antimicrobial resistance.

The pooled prevalence of any detected AMR carriage or infection among refugees and asylum seekers (33·0%, 95% CI 18·3–47·6; *I*^2^ =98%) was higher than it was in other migrants (6·6%, 1·8–11·3; *I*^2^ =92%; figure 4; table 2). In subanalyses by drug-resistant organism, the pooled prevalence of MRSA in refugees and asylum seekers (8·2%, 5·0–11·3; *I*^2^ =84%) was slightly higher than in other migrants (6·0%, 1·3–10·7; *I*^2^ =94%). The pooled prevalence of detected drug-resistant Gram-negative bacteria was similar in refugees and asylum seekers (27·2%, 17·2–37·1; *I*^2^ =95%) and other migrants (27·3%, 6·0–6·1; table 2), although this was only reported in one study.

**Figure 5:**
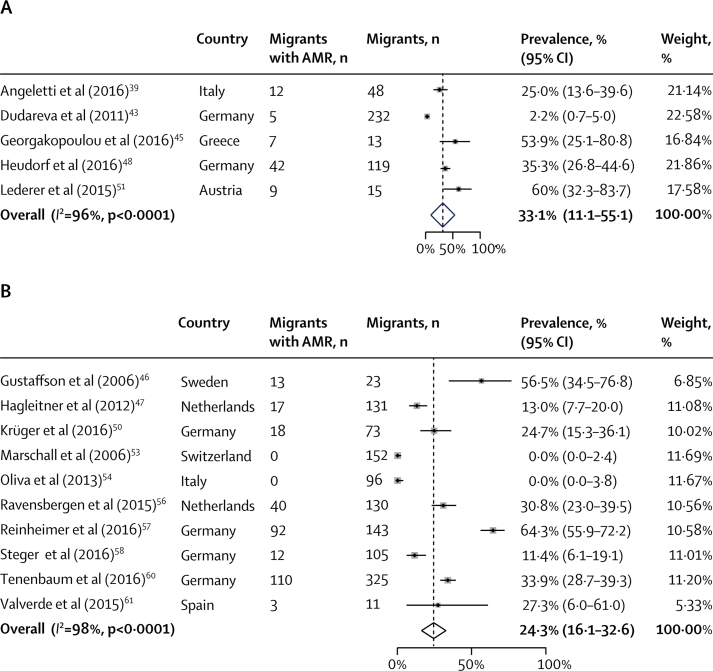
Pooled prevalence of antibiotic resistance by setting Prevalence in high-migrant settings (A) and in hospital settings (B). AMR=antimicrobial resistance.

The pooled prevalence of detected AMR carriage or infection in high-migrant community settings (33·1%, 95% CI 11·1–55·1; *I*^2^ =96%) was slightly increased compared with hospital settings (24·3%, 16·1–32·6; *I*^2^ =98%; figure 5; table 2). In subanalyses by drug-resistant organism, pooled prevalence of drug-resistant Gram-negative bacteria in high-migrant community settings (32·1%, 19·9–44·4; *I*^2^ =84%) was slightly higher than it was in hospital settings (24·6%, 9·5–39·8; *I*^2^ =97%; table 2). This was also true for MRSA in high-migrant community settings (9·8%, 0·0–20·3; *I*^2^ =94%) compared with in hospital settings (7·4%, 4·2–10·6; *I*^2^ =93%; table 2).

Migrants were also over-represented among individuals with community-associated MRSA, accounting for 62·7% (95% CI 50·2–75·3; *I*^2^ =12%) of Panton-Valentine leucocidin-positive MRSA isolates (a marker for community-associated MRSA^40^, ^41^, ^42^, ^44^, ^52^, ^62^, ^63^) reported among migrants and non-migrants in the included studies.^40^, ^41^, ^42^, ^44^, ^52^ Additionally, evidence from the included studies suggests that antibiotic-resistant organisms were being acquired during the migration trajectory in transit or host countries. In three articles, migrants with similar migration trajectories were colonised with the same microorganisms,^39^, ^45^, ^51^ and in one cross-sectional study in four refugee centres in Switzerland,^55^ MRSA clusters with recent transmission events and no relatedness in relation to regions of origin or travel routes were identified. Across the included articles, we did not find evidence of high rates of onward transmission to host populations.

## Discussion

Our systematic review and meta-analysis shows that the prevalence of AMR carriage and infection is elevated in refugees and asylum seekers and in high-migrant community settings in the included studies. Furthermore, evidence indicates that antibiotic-resistant organisms are being acquired by migrants during the migration trajectory in transit or host countries, suggesting that transmission of AMR is occurring during or following migration, either from local populations to migrants or between migrants. We did not find evidence of onward transmission by migrants to host populations, which might be partly attributable to social segregation.

Migrants' risk of exposure to antibiotic-resistant organisms in transit or host areas can be considered in the context of increased prevalence of AMR in high-migrant receiving countries, such as Greece (MRSA in 39·2% of tested clinical isolates and combined resistance for *E coli* and *K pneumoniae* in 10·7% of tested isolates), Italy (MRSA 34·1%, combined resistance 18·6%), and Spain (MRSA 25·3%, combined resistance 5·5%); compared with countries with a lower migrant intake, such as Sweden (MRSA 0·8%, combined resistance 2·4%).^64^ The effect that acquisition of AMR has in these countries—which might then be imported to northern European countries along the migration trajectory—should also be considered in the context of ongoing migration of EU migrants from southern to northern Europe and the implications that this situation could have for European countries with a low incidence of AMR.

In particular, our findings highlight the important role that high-migrant settings, including refugee camps, reception centres, transit centres, or detention centres, in transit and in host countries might have for the dissemination of drug resistance. Poor social conditions in these settings, such as inadequate sanitation, overcrowding, and restricted access to health services (including antibiotics or vaccinations), favour the spread of antibiotic-resistant infections.^13^, ^65^, ^66^ Such factors might be more influential determinants of AMR in migrants to Europe—especially refugees and asylum seekers—than the importation of these infections from countries of origin. In the case of MRSA, for example, colonisation with the same microorganisms has been seen in migrants with similar trajectories^39^, ^45^, ^51^ and clusters with recent transmission events.^55^ Migrants were also over-represented among Panton-Valentine leucocidin-positive MRSA cases, which are correlated with community-acquired infection.^40^, ^41^, ^42^, ^44^, ^52^, ^62^, ^63^ In view of concerns about the rising incidence of community-associated MRSA in Europe^67^, ^68^ and the increased virulence and severity of infection associated with Panton-Valentine leucocidin,^55^, ^63^ these findings highlight the need to improve screening and treatment of MRSA among refugees and asylum seekers in high-migrant community settings.

The prevalence of antibiotic-resistant Gram-negative bacteria is also increasing in community settings. For example, carriage of ESBL-producing enterobacteria has increased worldwide, including in community populations in Europe.^65^ Although carriage of community ESBL-producing enterobacteria is typically below 5% in Europe, elevated rates have been reported, including in Belgium (11·6%) and Spain (7·4%).^65^ Rising rates of carriage of antibiotic-resistant bacteria in healthy community populations have resulted in calls for improved antibiotic stewardship and prevention and control measures for infection in community settings. The elevated prevalence of AMR carriage in refugees and asylum seekers (33·0%), and in high-migrant community settings (33·1%), reinforce the need for such strategies.

The importance of high-migrant community settings (eg, camps, transit centres, or detention facilities) for the spread of infection has been highlighted in previous research,^40^, ^65^, ^66^ and whole-genome sequencing of drug-resistant strains of bacteria in refugees to Europe has shown that such organisms are being acquired in transit or host countries.^69^ Evidence that such settings contribute to the emergence of AMR has resulted in a call for improved prevention efforts in community settings,^7^ including by the European Centre for Disease Prevention and Control,^70^, ^71^ who have highlighted high rates of antibiotic-resistant bacteria in high-migrant settings (such as camps) and called for improved hygiene and targeted interventions to prevent the spread of antibiotic resistance in such locations.^71^

Routine testing for the carriage of antibiotic resistance is mainly done in hospitals, with a particular emphasis on targeted screening of high-risk patients at hospital admission (eg, recent antibiotic usage, recent contact with high-incidence countries, or having been admitted to hospital where AMR is endemic),^72^ although measures for the prevention and control of infection in these settings across Europe have been shown to vary substantially between and within countries.^73^ In many cases, protocols for screening and control of infectious disease transmission in these settings focus on patients who have come from or have been admitted to hospitals in countries with a high prevalence of drug-resistant organisms.^49^, ^57^, ^72^ However, migrants across the included articles had infrequently been admitted to hospital or had exposure to antibiotics, with evidence that AMR might have been acquired during or following migration. Furthermore, key emerging bacteria of concern in health-care settings, such as carbapenem-resistant Enterobacteriaceae, might be more prevalent among travellers who have had contact with health care in high prevalence countries (including European travellers or settled migrants in Europe making return visits to their countries of origin) than in those migrating to Europe.^74^ This hypothesis is consistent with literature demonstrating the particular risk of AMR acquisition and subsequent carriage in travellers to high prevalence countries.^9^

Such findings raise questions about the effectiveness or appropriateness of the current focus on screening in secondary care settings, and the targeting of migrants and refugees in particular. This uncertainty is reflected in recent calls for more robust evidence to guide policies regarding microbiological screening. Further research is needed to identify where and when migrants are acquiring AMR and, in terms of screening, what role it should play, who should be targeted, and where and how frequently it should be done.^60^, ^73^, ^75^, ^76^ The gap in evidence regarding the association between migration and AMR and effective infection prevention and control also aligns with a demand for more harmonised and evidence-based measures for the prevention and control of infection across Europe that are tailored to the local context and characteristics of at-risk groups.^73^ The increased rates of antibiotic resistance in high-migrant community settings shown in this systematic review highlight the potential benefit of screening in community settings rather than only in hospitals, especially in view of the substantial barriers that migrants can experience in accessing secondary health care.^39^, ^47^, ^57^

In addition to improved detection of AMR, for example through screening for colonisation, and improved antibiotic susceptibility testing of infections in high-migrant community settings, interventions should focus on better sanitation (for staff in migrant centres and the migrant population), increased quality of living conditions and less overcrowding, and better access to health services. These improvements will enable timely detection and treatment of infections when they do occur, thus reducing transmission and poorer and more costly health outcomes. Such initiatives could also be accompanied by health education and advocacy for migrants and relevant staff to increase awareness and improve standards of practice.

The findings provide compelling evidence for basic infection prevention and control, antibiotic surveillance principles in all aspects of health care,^73^ and targeted initiatives to improve detection and treatment of drug-resistant organisms in migrants in community settings. These changes should be accompanied by increased provider awareness and comprehensive risk assessments when engaging with migrant patients in relation to contact with health services, antibiotic use, or pre-migration risk factors, travel and immigration history, and exposure to poor social conditions.^75^, ^77^ Such strategies also need to be supported by improved access to health services and social conditions in these communities to reduce the risk of infection and transmission. This approach aligns with global frameworks, including the WHO Global Antimicrobial Resistance Surveillance System,^78^ the WHO draft framework of priorities and guiding principles to promote the health of refugees and migrants,^79^ and calls for explicit migrant health policies to address inequalities.^80^

In addition to improvements in the prevention, detection, and treatment of AMR in migrants, it is also important to reduce the stigma associated with these communities in view of the findings that migrants might acquire antibiotic-resistant organisms in host countries, and that the risk of onward transmission to host populations is low. This view is consistent with literature highlighting the continued vulnerability of migrants to infection in host countries, which is also seen for other infectious diseases like tuberculosis,^81^, ^82^, ^83^, ^84^ as opposed to the potential threat they present for transmission to host populations.^85^

Although our systematic review and meta-analysis addresses a key gap in the evidence base by identifying and synthesising data about patterns of antibiotic resistance in migrants to Europe, we acknowledge that the evidence has some key limitations. It is unclear how representative the migrants included in the studies are of the wider migrant population across Europe. Refugees and asylum seekers are over-represented and more data are needed from diverse migrant groups, including family reunion migrants, economic migrants, seasonal migrants, undocumented migrants, or settled migrants visiting their countries of origin. This over-representation might also have resulted in inflated prevalence estimates. A further limitation is that, in some studies, details about the reason for migration (eg, refugee or asylum seeker status) were not collected or reported, and thus could not be disaggregated in subanalyses. Data were heterogeneous, which contributed to variations in findings. Study outcomes, in particular, varied widely because of differences in definitions used, organisms and types of drug resistance reported, and measures (eg, carriage *vs* infection, sample prevalence or incidence, or proportion of drug-resistant isolates). Small sample sizes also contributed to wide confidence intervals in some subanalyses. To achieve a robust evidence base on AMR in migrant populations worldwide, efforts should be made to strengthen global surveillance systems, consistency in reporting, and data collection on migrant patients.

This systematic review and meta-analysis highlights the risk of AMR carriage and infection faced by migrants, particularly refugees, asylum seekers, and those residing in high-migrant community settings. The elevated prevalence of AMR in these groups could be attributed to the conditions they are exposed to in transit and host countries in Europe, which encourage the emergence and spread of drug resistance. The findings highlight a need for targeted strategies to improve the detection, treatment, and prevention of antibiotic resistance in high-migrant community settings, with little evidence to suggest that migrants significantly contribute to the burden of antibacterial resistance in Europe. Such strategies could include the development of Europe-wide screening guidelines, antimicrobial stewardship programmes in high-migrant community settings, and innovative approaches, such as technology-based surveillance or patient-held electronic records.^68^, ^86^ These approaches should be accompanied by integration of robust infection prevention and control as core practices throughout health services to prevent transmission of infection and development of antibiotic resistance. Such approaches are prioritised in the WHO global action plan on antimicrobial resistance^2^ and are particularly salient because of the barriers to accessing health care faced by migrants,^87^, ^88^ which are exacerbated by increasingly restrictive health services across Europe.^22^, ^89^ Our findings highlight that these initiatives need to be supported by strategies to minimise social deprivation, improve living conditions in high-migrant settings (such as refugee camps, transit centres, and detention facilities) in host countries in Europe, and improve—rather than restrict—access to high-quality health services for migrant communities regardless of legal resident status.^90^
