# Supplementary material for: Antimicrobial resistance among migrants in Europe: a systematic review and meta-analysis
Source: Lancet Infect Dis. 2018 Jul;18(7):796–811. doi: 10.1016/S1473-3099(18)30219-6 (PMC6032478; doi:10.1016/S1473-3099(18)30219-6)
Supplement: Supplementary appendix [file mmc1.pdf]

THE LANCET Infectious Diseases

Supplementary webappendix

This webappendix formed part of the original submission and has been peer reviewed. We post it as supplied by the authors.

Supplement to: Nellums LB, Thompson H, Holmes A, et al. Antimicrobial resistance among migrants in Europe: a systematic review and meta-analysis. *Lancet Infect Dis* 2018; published online May 17. http://dx.doi.org/10.1016/S1473-3099(18)30219-6.

Antimicrobial resistance among migrants in Europe: a systematic review and meta-analysis

Laura B. Nellums PhD*[a], Hayley Thompson MPH*[a], Professor Alison Holmes FMedSci[a], Enrique Castro-Sánchez PhD[a], Jonathan A. Otter, PhD[a], Marie Norredam MD[b], Professor Jon S. Friedland FMedSci[a], Sally Hargreaves FRCPE[a],

*Joint first authors

[a] Department of Medicine, Imperial College London, London, United Kingdom

[b] Danish Research Centre for Migration Ethnicity and Health, University of Copenhagen, Copenhagen, Denmark & Section of Immigrant Medicine, Department of Infectious Disease, Copenhagen University Hospital, Hvidovre, Denmark.

Correspondence to:

Dr. Sally Hargreaves

8th Floor Commonwealth Building, International Health Unit, Infectious Diseases & Immunity, Imperial College London, Hammersmith Hospital, Du Cane Road, London W12 ONN UK

s.hargreaves@imperial.ac.uk

Supplementary table 1 Boolean search strategy

**Keywords for database search**	(migrant* OR migration OR immigra* OR refugee* OR asylum OR foreign born OR foreign-born) AND (microbial drug resistan* OR bacterial resistan* OR antibiotic resistan* OR antimicrobial resistan* OR XDR OR MDR OR multi-drug resistan* OR extensively drug-resistan* OR extensively drug resistan* OR drug resistan* OR drug-resistan* OR antimicrobial resistan* OR resistan* OR non-susceptib* OR decreased susceptibility OR beta-lactamase* OR extended-spectrum beta-lactamase* OR ESBL OR CTX-M OR AmpC OR metallo-beta-lactamase* OR MBL OR methicillin-resistant Staphylococcus aureus OR MRSA) AND (bacteri* Escherichia OR E coli OR Klebsiella* OR Staphylococcus OR Streptococcus OR Salmonell* OR Shigell* OR Gonorrhoeae OR gonorrhea OR Proteus OR Enterobacter* OR Morganella OR Tuberculosis OR TB OR Yersinia* OR Gram negative OR Fluoroquinolon* OR Cephalosporin* OR Carbapenem* OR Penicillin) AND (European Union OR EU OR European Economic Area OR EEA OR European Free Trade countr* OR Austria OR Belgium OR Britain OR Czech Republic OR Denmark OR England OR Finland OR France OR Germany OR Greece OR Ireland OR Iceland OR Italy OR Lichtenstein OR Luxembourg OR Netherlands OR Norway OR Portugal OR Scotland OR Spain OR Sweden OR Switzerland OR United Kingdom OR UK OR Wales)
**Search strategy with MeSH headings for Medline database search**	(Emigration and Immigration OR Refugees OR Emigrants and Immigrants OR Human Migration OR Transients and Migrants) AND (Bacterial Infections OR Drug Resistance, Bacterial OR Anti-Bacterial Agents OR Drug Resistance, Microbial OR Extensively Drug-Resistant Tuberculosis OR Tuberculosis, Multidrug-Resistant OR Drug Resistance, Multiple OR Drug Resistance, Multiple, Bacterial OR Drug Resistance OR beta-Lactamases OR Methicillin-Resistant Staphylococcus aureus OR Methicillin Resistance) AND (Escherichia coli OR Escherichia OR Klebsiella Infections OR Klebsiella OR Staphylococcus OR Streptococcus OR Salmonella OR Salmonella Infections OR Shigella OR Gonorrhea OR Neisseria gonorrhoeae OR Proteus OR Enterobacter OR Enterobacteriaceae OR Morganella OR Yersinia OR Gram-Negative Bacterial Infections OR Gram-Negative Bacteria OR Fluoroquinolones OR Quinolones OR Cephalosporins OR Penicillins OR Carbapenems) AND (European Union OR Austria OR Belgium OR England OR Great Britain OR Czech Republic OR Denmark OR Finland OR France OR Germany OR Northern Ireland OR Ireland OR Italy OR Luxembourg OR Netherlands OR Switzerland OR Norway OR Iceland OR Portugal OR Scotland OR Spain OR Sweden OR Wales)

Citation	Country	Migrant type	MRSA (n)	N	Prevalence (% [95% CI])	Weight
Angeletti, 2016	Italy	Asylum seekers	6	48	12.5 [4.7 – 25.3]	4.94
Dudareva, 2011	Germany	Asylum seekers	5	232	21.6 [0.7 – 5.0]	9.60
Gustaffson, 2006	Sweden	Adopted children	13	23	56.5 [34.5 – 76.8]	1.72
Hagleitner, 2012	Netherlands	Adopted children	17	131	13.0 [7.7 – 20.0]	7.21
Heudorf, 2016b	Germany	Refugees	32	325	10.0 [6.8 – 13.6]	8.90
Krüger, 2016	Germany	Refugees	11	73	15.1 [7.8 – 25.4]	5.59
Marschall, 2006	Switzerland	Migrant women	0	152	0.0 [0.0 – 2.4]	9.90
Oliva, 2013	Italy	HIV-negative migrants	0	96	0.0 [0.0 – 3.8]	9.76
Piso, 2017	Switzerland	Refugees	41	261	15.7 [11.5 – 20.7]	8.14
Ravensbergen, 2015	Netherlands	Asylum Seekers	10	130	7.7 [3.8 – 13.7]	8.03
Reinheimer, 2016	Germany	Refugees	8	143	5.6 [2.5 – 10.7]	8.57
Steger, 2016	Germany	Asylum Seekers	4	96	4.2 [1.2 – 10.3]	8.42
Tenenbaum, 2016	Germany	Refugee children	22	325	6.8 [4.3 – 10.1]	9.19
Overall (I^2 = 92.7089%, p = 0.0000)					7.8 [4.8 – 10.7]	100.00

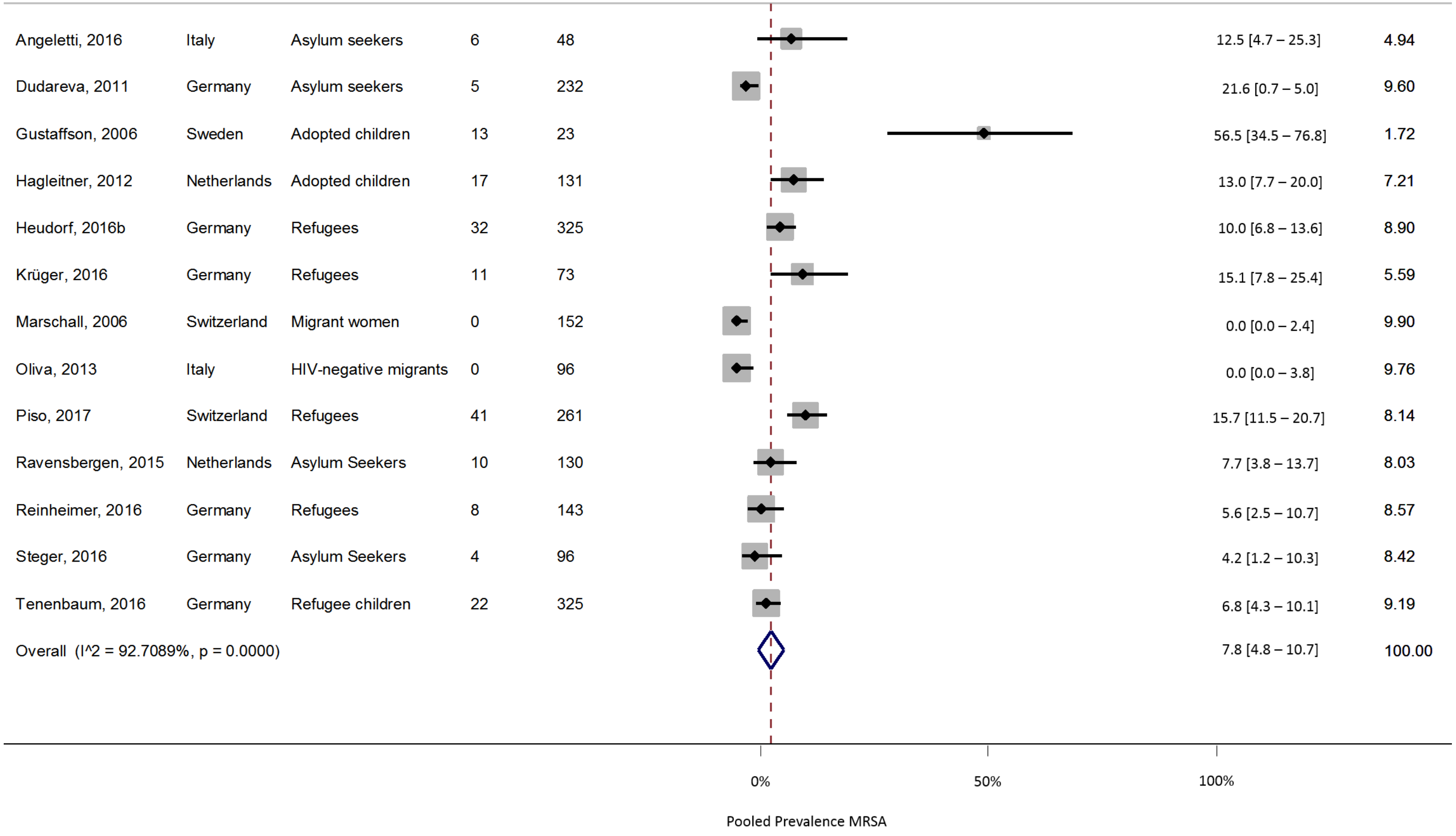

Supplementary figure 1 Pooled prevalence of MRSA

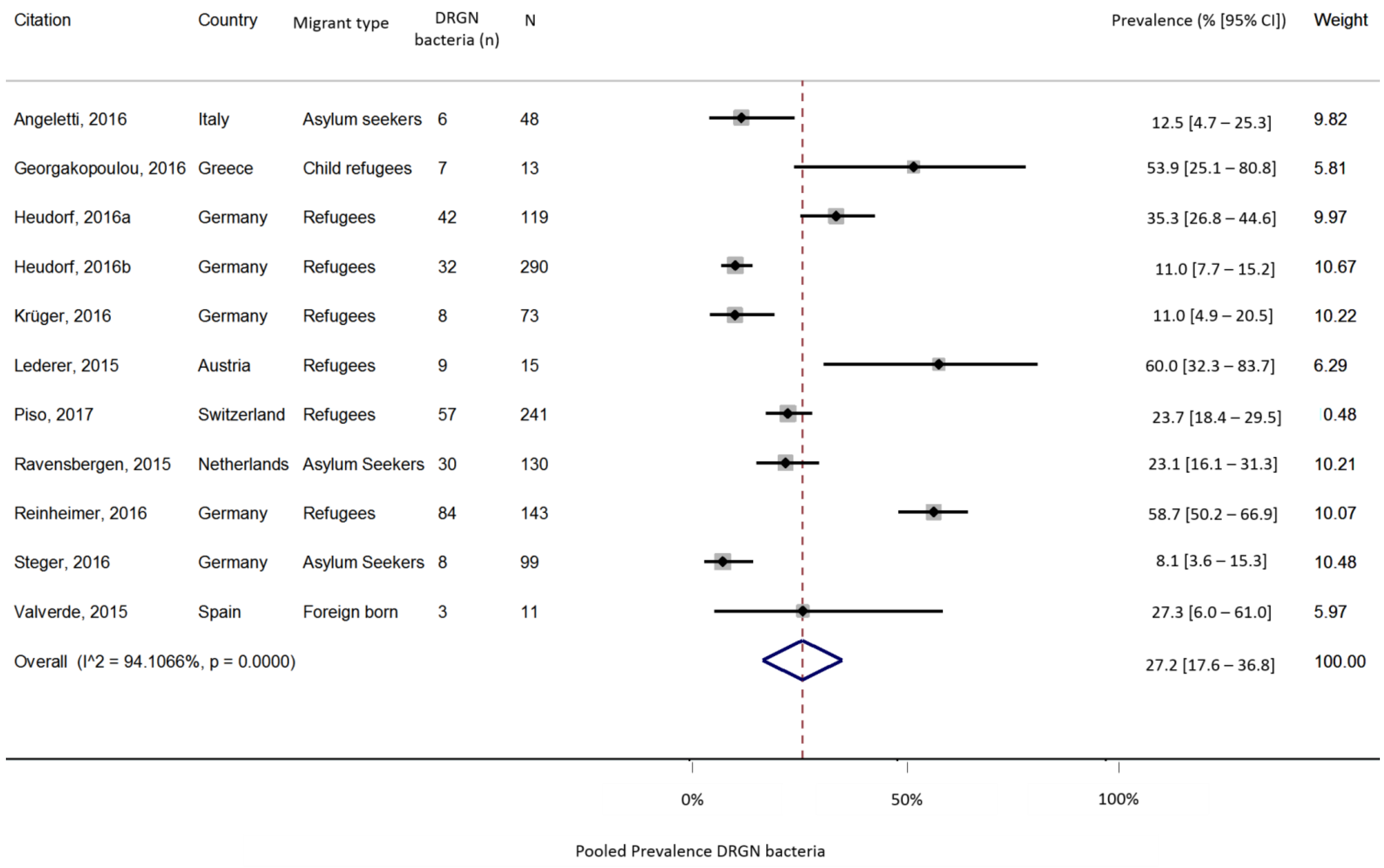

Supplementary figure 2 Pooled prevalence of drug resistant Gram-negative bacteria

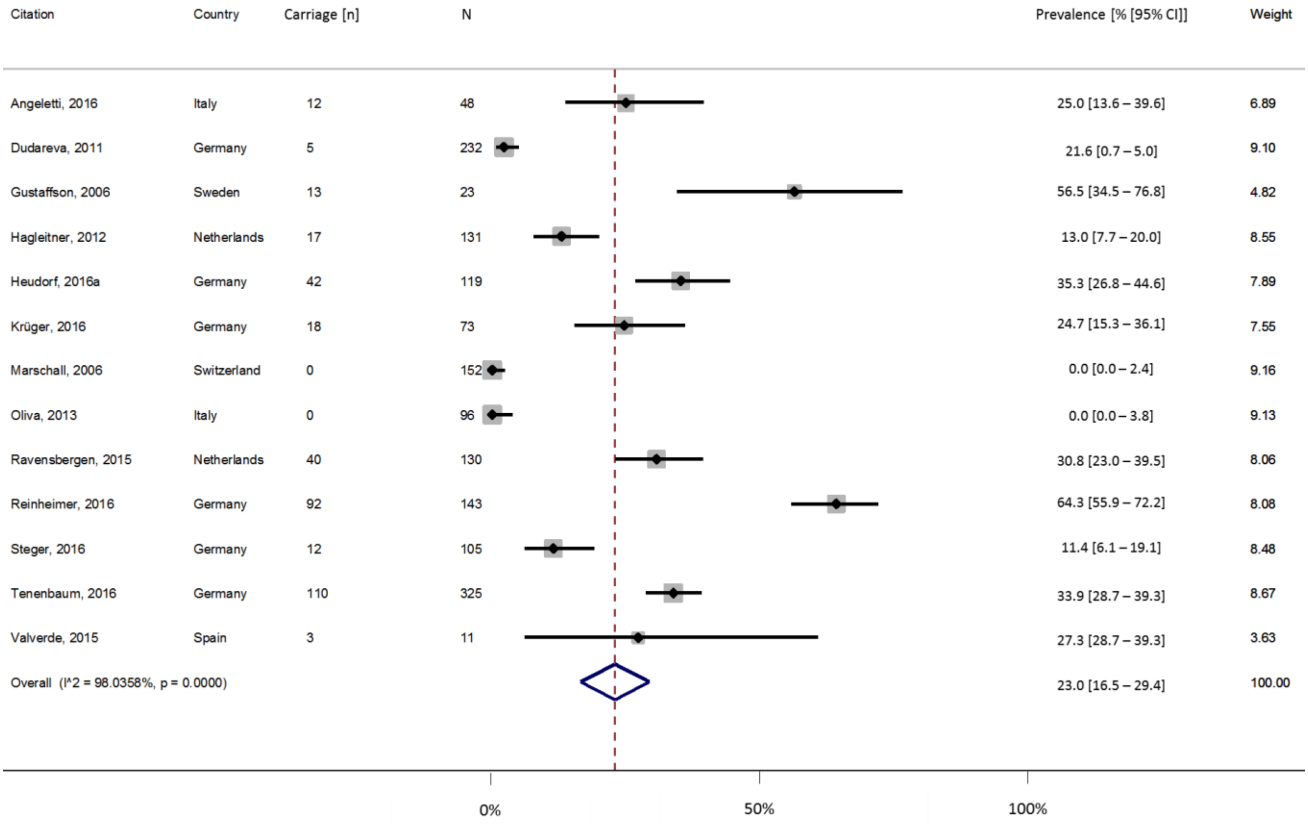

Supplementary figure 3 Pooled prevalence of detected AMR carriage

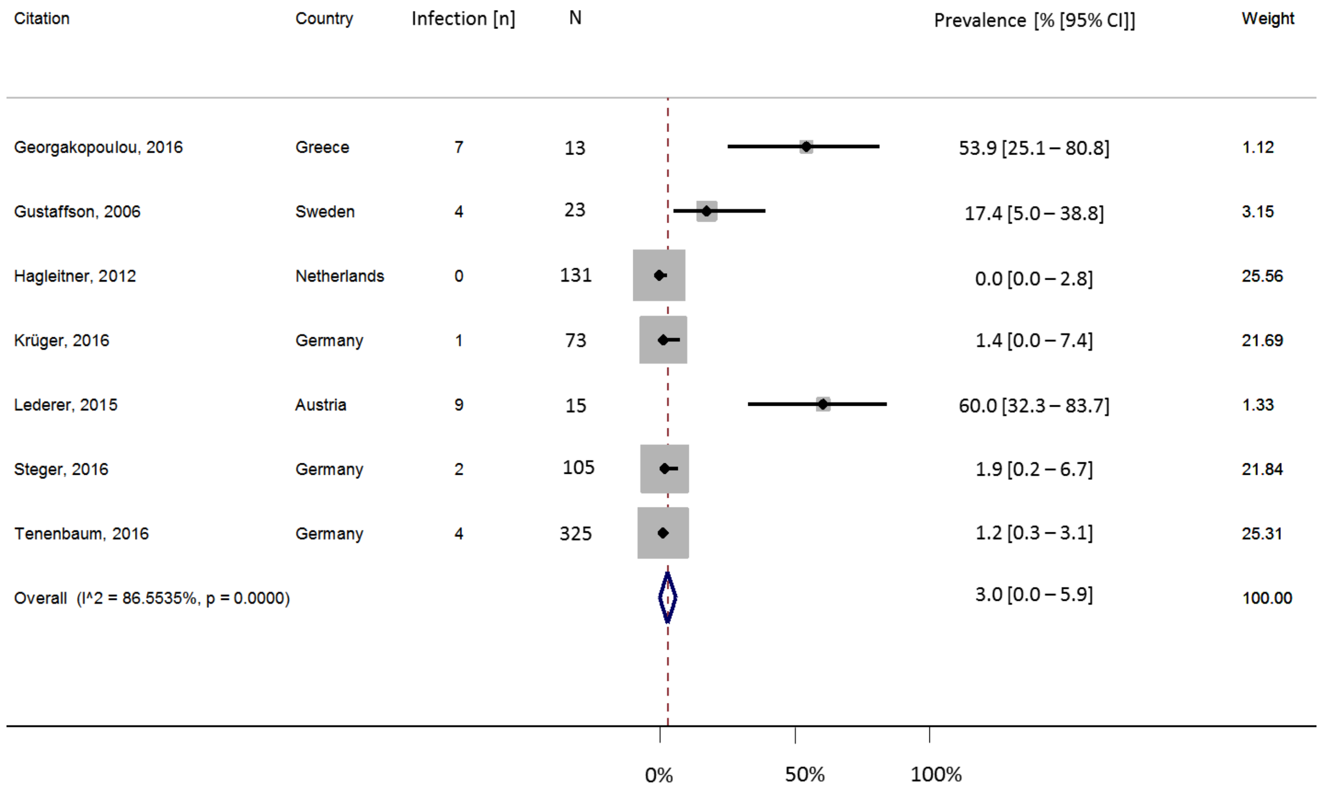

Supplementary figure 4 Pooled prevalence of detected AMR infection
